# A Culturally Tailored Decision Aid to Support Dementia Care Planning Among Chinese American Family Caregivers

**DOI:** 10.1093/geroni/igaf122.844

**Published:** 2025-12-31

**Authors:** Chien-Ching Li, Tingfei Hu, Sharon Mei, Samantha Wu, Olimpia Paun

**Affiliations:** Rush University, Chicago, Illinois, United States; Rush University, Chicago, Illinois, United States; University of Illinois Chicago, Chicago, Illinois, United States; University of Illinois Chicago, Chicago, Illinois, United States; Rush University, Chicago, Illinois, United States

## Abstract

The Chinese American population, the largest Asian subgroup in the U.S., is aging rapidly. Consequently, Chinese American family caregivers of individuals with dementia face increasing challenges in healthcare planning. We have developed a culturally tailored, web-based decision aid to help caregivers make informed choices about home and long-term care. The tool provides essential information on dementia symptoms across stages, care options, costs, insurance coverage, and key decision factors. It also includes a Self-Assessment that generates a personalized summary to guide caregivers’ decision-making process. In partnership with two Chinese community organizations in Chicago, we recruited a total of n = 10 Chinese American family caregivers of loved ones living with dementia or potential dementia symptoms for the study. Participants were asked to use the web-based DA tool and complete usability and acceptability surveys. Descriptive analysis was conducted to describe the usability and acceptability testing results. Results indicated that study participants were highly satisfied with the DA tool. Key feedback reported by the majority of participants includes: (1) the length and amount of information presented in the tool was just right, (2) this tool can provide sufficient information to make appropriate care decisions for loved ones with dementia., (3) various functions in this tool are well integrated, and (4) I will frequently use this tool to help me discuss dementia care plans and decisions with family and providers. Future efforts should focus on developing and implementing interventions that integrate the web-based decision-aid tool to better support Chinese American family caregivers.

